# A Study of the Kinematics System in Drilling Inconel 718 for Improving of Hole Quality in the Aviation and Space Industries

**DOI:** 10.3390/ma15165500

**Published:** 2022-08-10

**Authors:** Mateusz Bronis, Edward Miko, Lukasz Nowakowski, Marian Bartoszuk

**Affiliations:** 1 Department of Manufacturing Engineering and Metrology, Kielce University of Technology, al. Tysiaclecia Panstwa Polskiego 7, 25-314 Kielce, Poland; 2 Department of Manufacturing and Materials Engineering, Opole University of Technology, ul. S. Mikołajczyka 5, 45-271 Opole, Poland

**Keywords:** drilling, universal turning center, Inconel, hole quality, form errors, surface texture, ANOVA

## Abstract

This article discusses experimental results concerning the quality of through holes drilled in Inconel 718. The tests involved hole cutting under 27 different conditions using different values of the feed per revolution and spindle speed, and different types of kinematic system. The drilling was performed on a CTX Alpha 500 universal turning center using tools with internal coolant supply. Three kinematic systems were considered for hole cutting. The first, based on the driven tool holder, had a stationary workpiece and a rotating and linearly fed tool. In the second, where drilling was based on the spindle rotations, the workpiece rotated while the tool moved along a straight line. In the third system, the workpiece and the tool rotated in opposite directions; the tool also performed a linear motion. The study aimed to assess the quality of holes on the basis of the following output parameters: the hole diameter, cylindricity and straightness errors, and the surface texture. A multifactorial statistical analysis was used to determine how the hole quality was dependent on the process parameters and the type of drilling kinematics. The findings confirm that the kinematic system, as well as the feed per revolution, are the key factors affecting the quality of holes drilled in Inconel 718. The analysis of the hole drilling process for Inconel 718, performed using a CNC turning center, shows that the third kinematic system was the best option as all the four parameters describing the hole quality had the lowest values. The best results were obtained in the 6th (*n* = 637 rpm, *f_n_* = 0.075 mm/rev, KIN III) and 8th experiments (*n* = 955 rpm, *f_n_* = 0.075 mm/rev, KIN II), because the parameters were then the lowest, with the scatter of results being up to 30%.

## 1. Introduction

Inconel 718 is a high-performance alloy based on nickel and chromium with a wide variety of applications, mainly in the aviation and space industries. Due to its low thermal conductivity and high strength at high temperatures, as well as other excellent properties, the alloy can be used under different environmental conditions [1]. Despite the fact that the material is difficult to shape and machine, as it tends to harden at the surface during cutting, it can be fabricated even into complex parts [2]. In the aviation industry, drilling in Inconel 718 constitutes about 50% of all machining operations [3].

The research on the hole drilling in Inconel 718 has been quite extensive; there are many studies dealing with process-related problems, especially the surface quality. Khanna et al. [4], for example, investigated how the cooling/lubrication conditions (dry and cryogenic drilling) affected the quality of holes cut in this alloy, i.e., their cylindricity, circularity, and surface roughness R_a_. The process parameters, however, were kept constant. The test results indicate that, under cryogenic drilling conditions, the surface roughness parameter, R_a_, drops to 47% when compared to that obtained in dry drilling. In [5], Ahmed et al. observe that the straightness error changes depending on the coolant pressure and the spindle speed. They suggest that the higher the cooling pressure and the spindle speed, the lower the hole straightness error. Oezkaya et al. [6] analyze the effects of internal and external cooling. The parameters studied are the hole straightness error and the surface roughness parameter R_z_. The lowest values of the parameter R_z_ and the hole straightness error were obtained at 60 bar internal coolant supply. This type of cooling eliminated the dead zones near the cutting edge. In [7], Sharman et al. consider five different geometries of the drill bit. The purpose of their study was to establish whether the tool geometry had any effect on the surface roughness parameter R_a_ in hole cutting. The lowest values of the parameter R_a_ were observed for a CS tool with a curved cutting edge and a sharp corner. Uçak and Çiçek [8] provide extensive analysis of the drilling process for two different types of drill bits (uncoated and TiAlN coated) and three different cooling conditions (dry, cryogenic, and wet drilling); they show the influence of these factors on the hole diameter, hole roundness, and the height of burrs at the hole entry and exit. The results indicate that cooling with LN_2_ helps reduce the roundness error by 20–69%, and the occurrence of burrs by 3–27% at the hole entry and by 15–54% at the exit; the machining of Inconel 718 under wet conditions is 30–56% more efficient in terms of surface roughness than during dry or cryogenic drilling. Neo et al. [9] focus on the hole quality, i.e., surface roughness, roundness error, measured every 50 mm, and straightness error, obtained at four different values of the spindle speed (1500, 2000, 2500 and 4500 rpm). They found that the highest spindle speed they used resulted in the lowest values of the output parameters. Karabulut and Kaynak [10] analyze how different values of the feed per revolution (0.025, 0.05 and 0.075 mm/rev) and cutting speed (15 and 30 m/min) are responsible for the surface roughness, described by the parameter R_a_. They conclude that high cutting speeds and high feed rates result in the occurrence of scratches and debris at the hole surface. Müller et al. [11] offer an interesting approach; they study the surface roughness and roundness of holes in relation to the diameter (1, 1.4 mm), number (2, 4), shape (round, triangular), and angle (25 and 15 degrees) of the cooling channels. When drill bits with a greater diameter and a smaller angle of the cooling channel were used, the holes had the lowest surface roughness. The research described in [12,13] is concerned with the influence of the cutting speed, feed per revolution, and the type of kinematic system on the geometrical and dimensional accuracy of holes drilled in 42CrMo4 + QT steel and C45 steel. For 42CrMo4 + QT steel the first kinematic system is the most suitable, as 3 out of 4 parameters studied (CYL, STR, RON) reached the lowest values. However, for C45 steel, the lowest values of DE, STR and CYL were observed when the second kinematic system was used. In [14], Thrinadh et al. investigate how the cutting speed (65 and 85 m/min) and depth of cut (0.2 and 0.5 mm) affect the machinability of Inconel 718. They claim that the higher the cutting speed and the depth of cut, the higher the process temperature; this may lead to thermal cracking, plastic deformation and oxidation. Sahoo et al. [15] optimize the drilling process in terms of the tool wear, spindle speed (215, 315 and 455 rpm) and feed per revolution (0.106, 0.213, and 0.316 mm/rev) to obtain the lowest surface roughness. They found that at 455 rpm and 0.106 mm/rev, the surface roughness was the lowest. Shah et al. [16] study the hole cutting in Inconel 718 under cryogenic cooling conditions with LN_2_ and LCO_2_ at a constant feed of 0.045 mm/rev and a cutting speed of 10, 15 or 20 m/min. They show that the parameter R_a_ decreases by 11% under LCO_2_ cooling conditions when compared with LN_2_ cooling. The practical approach presented in [17] deals with decision making to enhance the hole drilling process. The research involved comparing different models developed over recent years. The simulations, including not only predictive modelling but also analysis of various interactions observed during the cutting process, aimed to improve the preparation stage. Sugiura et al. [18] confirm that hole drilling modeling and simulations are very important, as they help verify the results and avoid design errors.

From the review of the literature, it is apparent that there are no studies describing the combined effects of the process parameters and kinematics on the quality of holes drilled in Inconel 718. The novelty of this research is the multifactorial analysis of the influence of three different kinematic systems for drilling through holes in Inconel 718 using a CNC turning center. Most studies on hole drilling deal with one surface quality parameter, and such an approach seems insufficient. From the literature analysis, it can be concluded that the most important parameters describing the hole quality are: the cylindricity error; straightness error; roundness error; diameter error; surface roughness; and burrs. This article attempts to investigate how the hole cylindricity, straightness and diameter errors, as well as surface roughness (CYL, STR, DE, R_a_), are dependent on the process parameters and the type of kinematic system.

## 2. Materials and Methods

The purpose of this study was to determine how the feed per revolution, spindle speed and type of kinematic system affected the cutting of through holes in Inconel 718. Due to its high strength, high resistance to corrosion and high fracture toughness, the material is widely used, for instance, in nuclear reactors, pumps, rocket engines, spacecraft and gas turbines. In the oil and natural gas sector, it is also common, mainly due to its high corrosion resistance and tensile, creep, fatigue and rupture strength [17]. Table 1 shows the chemical composition of Inconel 718, while Table 2 lists its main properties.

**Figure 1:**
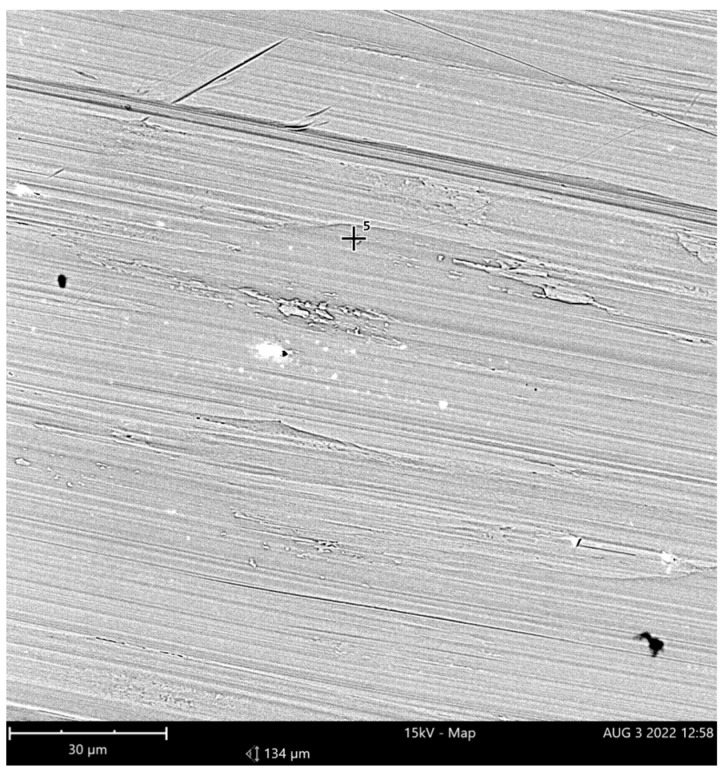
A SEM image of the material studied.

The actual composition, given in Table 1, was determined using a Phenom XL scanning electron microscope. Figure 1 depicts a SEM image of the hole drilled.

The tests were performed using a DMG MORI CTX Alpha 500 universal turning center. The experiments aimed to determine whether the hole quality was dependent on the main process parameters, i.e., the feed per revolution and spindle speed, as well as the type of kinematic system. Three kinematic systems were considered, as illustrated in Table 3.

The experiments were carried out using a carbide drill bit coated with titanium aluminum nitride, which allowed internal coolant supply. The process details are provided in Table 4. The key element of the three kinematic systems was a VDI30 SAUTER 113180 driven tool holder, which helped make the tool rotate. The tool was clamped using an ER25 DIN 5480 collet chuck.

### Design of Experiments

**Figure 2:**
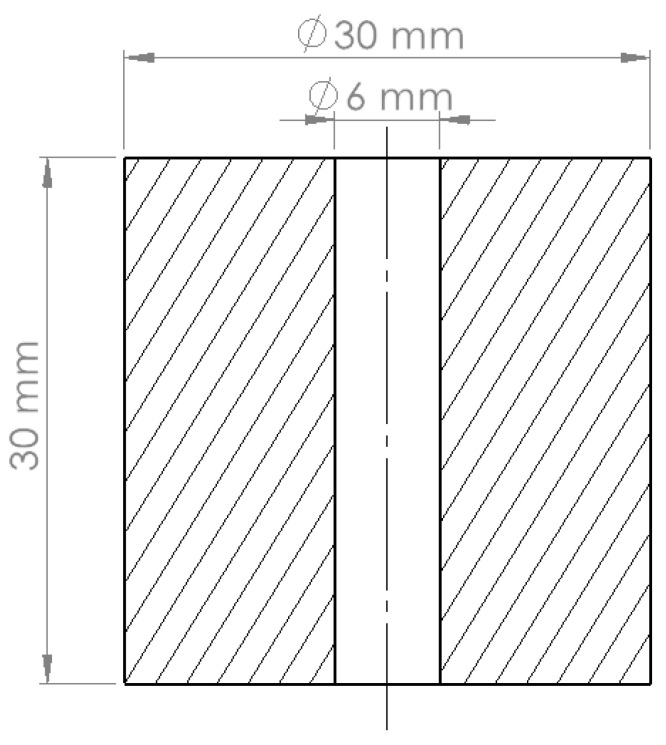
Drawing of the workpiece with a through hole.

The holes were drilled in 27 workpieces all with a diameter of 30 mm and a length of 30 mm. Before drilling, the top surface of each workpiece was polished by turning. Figure 2 shows the geometry of the workpieces used in the drilling tests. 

The ranges of the process parameters to be used in the tests were selected on the basis of the literature and the authors’ own studies. The experiments were conducted for different combinations of the input parameters (637; 800; 955 rev/min, 0.06; 0.075; 0.09 mm/rev, KIN I; KIN II; KIN III), as provided in Table 5. The data shown in Table 5 were then used in the statistical analysis. The kinematics of the drilling process was written as Equation (1), describing the resultant rotational speed.

(1) $$KIN=n_{n}-n,$$

where: *KIN*—kinematic system, *n_n_*—tool speed, *n*—spindle speed.

**Figure 3:**
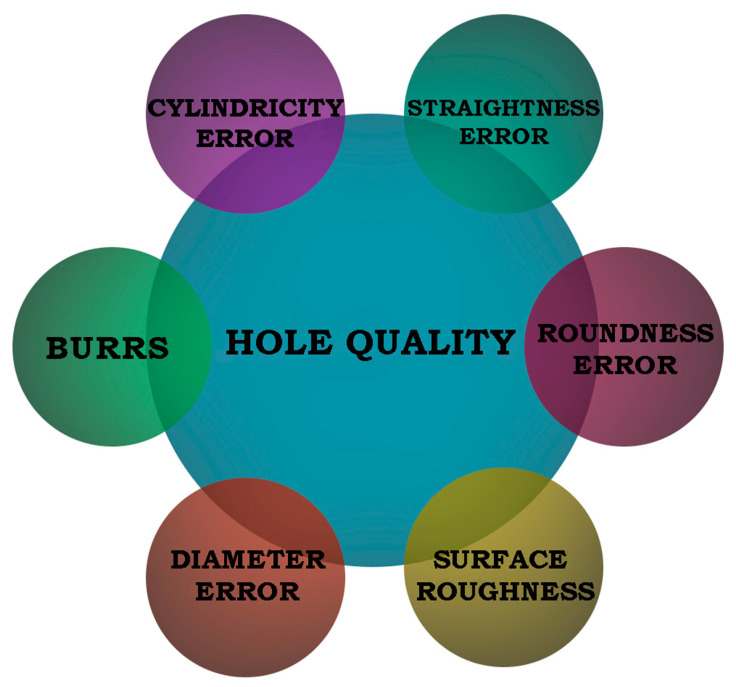
Parameters describing the hole quality.

The diagram in Figure 3, developed on the basis of a review of the literature, shows the key parameters describing the hole quality. In this study, four out of six were analyzed: diameter error; surface texture; straightness error; and cylindricity error.

**Figure 4:**
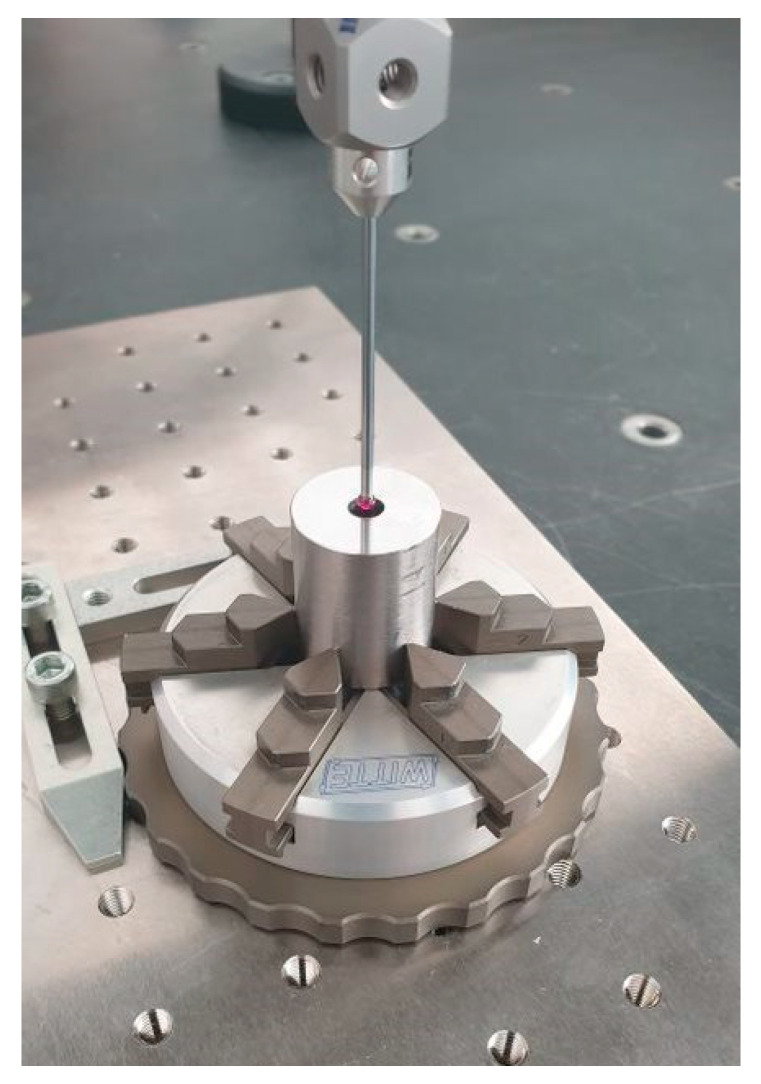
Workpiece clamping in the PRISMO Navigator coordinating machine.

**Figure 5:**
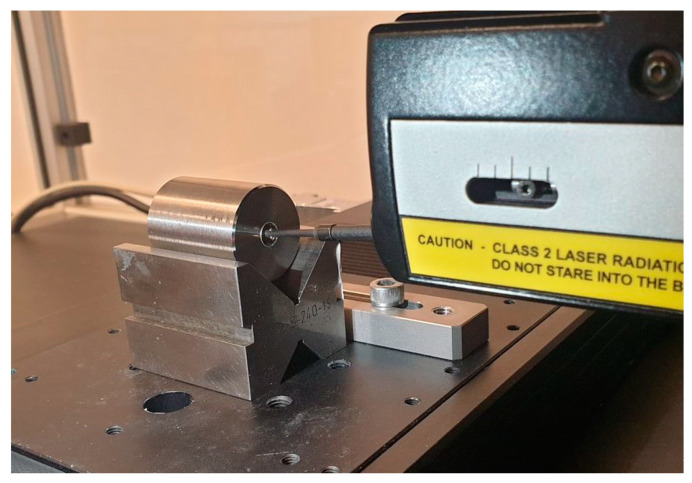
Workpiece clamping in the Form Talysurf PGI 1230.

The hole diameter, cylindricity and straightness errors were measured using a ZEISS PRISMO Navigator (Figure 4) coordinate measuring machine at the Department of Manufacturing Engineering and Metrology of the Kielce University of Technology. The machine features excellent dynamics, high speed with maximum precision, outstanding resistance to ambient conditions, high rigidity, and passive vibration damping with elastomer spring elements [12]. The measurements were taken at a speed of 5 mm/s using a ruby probe stylus ball tip with a radius of 1.5 mm. The cylindricity error was determined on the basis of five cross-sections by applying the roundness profile strategy. The measurements were carried out with a 15 UPR Gaussian filter (λc = 2.5 mm) in accordance with the standards concerning the ratio of the reference circle diameter to the probe tip radius. The surface texture of the holes was measured using a Form Talysurf PGI 1230 (Figure 5) surface finish system by Taylor Hobson. The system, equipped with a laser interferometer, is suitable for high precision 2D and 3D measurement of surface texture, offering a measurement resolution of 0.8 nm, a runout of less than 1 µm, and a gauge range of 12.5 mm, a straightness accuracy of less than 0.2 µm, and a traverse length of 200 mm. The Form Talysurf PGI 1230 system was used to measure the arithmetic mean of the absolute ordinate within the sampling length (R_a_). This parameter was calculated along a sampling length of 0.8 mm. A Gaussian filter was used for this purpose (λc = 0.8 mm). The measurements were taken at a speed of 0.5 mm/s using a diamond stylus with a nose radius of 2 µm. The sampling step was 0.125 µm.

## 3. Results and Discussion

### 3.1. Metrological Analysis

**Figure 6:**
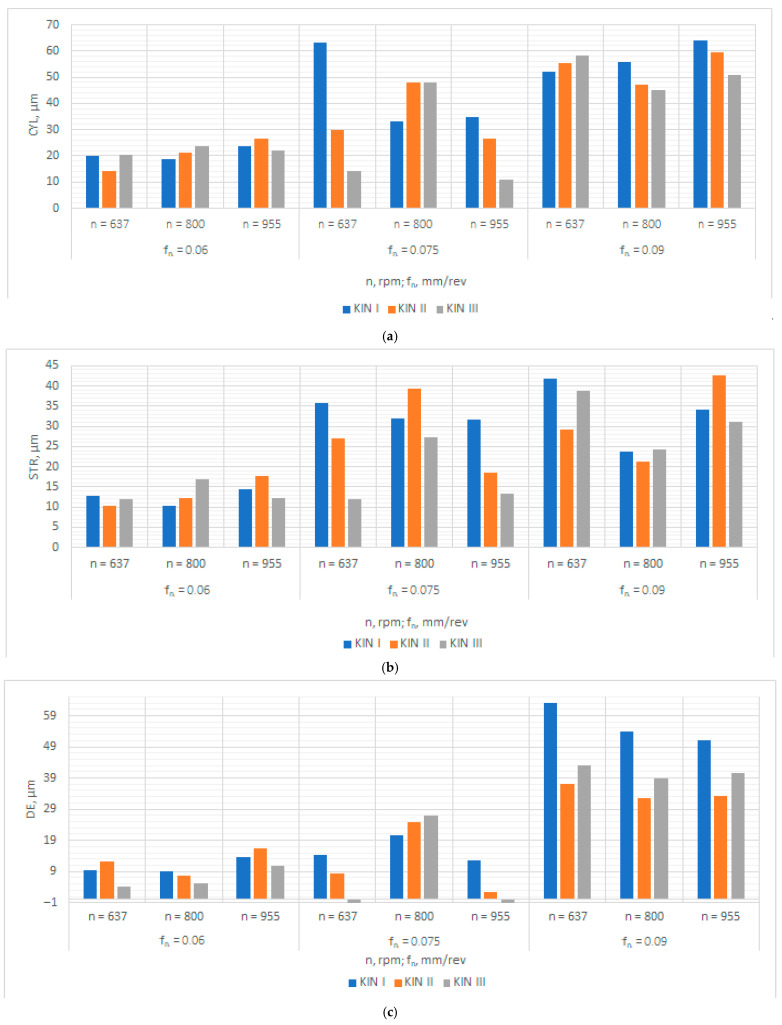

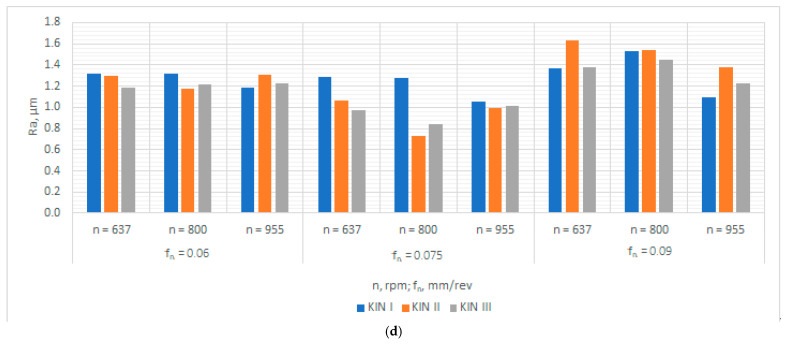
Graphical representation of the metrological analysis data for: (**a**) the cylindricity error; (**b**) the straightness error; (**c**) the diameter error; and (**d**) the parameter R_a_.

The metrological analysis helped look at the relationships between the input parameters (i.e., feed per revolution, spindle speed and type of kinematic system) and the output parameters (hole diameter error, surface texture, cylindricity error, and straightness error). The results are given in Figure 6. From Figure 6a, it is apparent that the lowest cylindricity error of 10.9 µm was obtained at *n* = 955 rpm, and *f_n_* = 0.075 mm/rev when the third kinematic system was used. The highest cylindricity error of 63.2 µm was reported for the first kinematic system at *n* = 637 rpm and *f_n_* = 0.09 mm/rev. As can be seen from Figure 6a, an increase in the feed per revolution led to an increase in the cylindricity error. The results concerning the straightness error are shown in Figure 6b. It is clear that the lowest value of this parameter (STR = 10.3 µm) was obtained for *n* = 637 rpm, *f_n_* = 0.06 mm/rev and the second kinematic system. The highest value of 42.7 µm was observed at the highest values of the process parameters (*n* = 955 rpm; *f_n_* = 0.09 mm/rev) and for the second kinematic system. At the lowest feed per revolution (0.06 mm/rev), the straightness error was the smallest. Figure 6c indicates that the most accurate hole was produced at the highest spindle speed (955 rpm) and a medium feed per revolution of 0.075 mm/rev when the third kinematic system was applied. The least accurate holes, on the other hand, were drilled at feeds per revolution of 0.06 and 0.075 mm/rev. The worst results were obtained at the highest feed per revolution (0.09 mm/rev). From Figure 6d, it is evident that the lowest value of the parameter R_a_ (0.727 µm) was obtained at medium values of the process parameters (*n* = 800 rpm; *f_n_* = 0.075 mm/rev) when the second kinematic system was employed. The highest surface roughness was observed for a feed per revolution of 0.09 mm/rev. Another finding is that the highest process efficiency was obtained at a medium feed per revolution of 0.075 mm/rev. In the multifactorial analysis, each input parameter was assigned a weight of 1.0; they were assumed to be equally important. Two sets of input parameters were selected because of the lowest values of the output parameters (CYL, STR, DE, R_a_). The most optimal conditions were reported for the 6th (*n* = 637 rpm, *f_n_* = 0.075 mm/rev, KIN III) and 8th experiments (*n* = 955 rpm, *f_n_* = 0.075 mm/rev, KIN II), because the parameters were then the lowest, with the scatter of results being up to 30%.

### 3.2. Statistical Analysis (ANOVA)

The experimental results were studied using the analysis of variance (ANOVA) to determine how each value obtained was dependent on different factors. The statistical analysis involved deriving Taguchi L27 orthogonal arrays. The purpose was to calculate and discuss the influence of the different values of the input parameters on the output parameters. The analysis was carried out using Statistica. Each analysis was performed at a confidence interval of 95% and a significance level of 5%. The response surface method was applied because of its hybrid nature; it combines polynomial and factorial (fractional) regression models. The results of the ANOVA statistical analysis, provided in Table 6, Table 7, Table 8 and Table 9, show the relationships between the input and output parameters.

The calculated values of SS and MS helped obtain the f value, on the basis of which the significance of the statistical analysis was read from the arrays. As can be seen from Table 6, Table 7, Table 8 and Table 9, all the models have values less than 0.05, which suggests the significance of the factors considered in the models; from these tables, it is evident that the feed per revolution was the major factor contributing to the diameter error (75.75%), R_a_ (75.36%) and the straightness error (49.69%). The cylindricity error (61.81%) was dependent mainly on the type of kinematic system used for the drilling. From Table 6, it is evident that the influence of the spindle speed on the cylindricity error reached 18.21%, while that of the feed per revolution was 19.36%. According to Table 7, the influence of the kinematic system on the hole straightness error was 32.83%, whereas that of the spindle speed was 7.41%. Table 8 indicates that the influence of the type of kinematic system on the hole diameter error reached 12.26%, while that of the spindle speed was 9.42%. From Table 9, it is clear that the kinematic system and the spindle speed had little influence on this parameter (7.19% and 2.32%, respectively). In contrast to other studies [12,13], this analysis shows that the hole cylindricity error was mainly dependent on the other process parameter studied, i.e., the feed per revolution. For Inconel 718, feed per revolution was the key factor affecting the hole straightness and diameter errors.

The response surface model equation was used to develop the regression models for the cylindricity error, the straightness error, the diameter error, and the surface roughness parameter R_a_. The empirically built regression models 2–5 were characterized by high correlation values. The coefficients of determination were: 70.49% for the cylindricity error, 62.13% for the straightness error, 88.50% for the diameter error, and 69.73% for the parameter R_a_. The values of the standard estimation error for each model were as follows: 9.4 for the cylindricity error, 6.7 for the straightness error, 6.2 for the diameter error, and 0.118 for the parameter R_a_.

(2) $$\mathrm{CYL}=-29.94+1.33\cdot 10^{-1}\cdot n-7.60\cdot 10^{-5}\cdot n^{2}-739.39\cdot f_{n}+13975.31\cdot f_{n}{}^{2}+1.12\cdot 10^{-2}\cdot KIN+9.27\cdot 10^{-6}\;\cdot KIN^{2}+3.24\cdot 10^{-1}\cdot n\cdot f_{n}-1.89\cdot 10^{-5}\cdot n\cdot KIN+8.84\cdot 10^{-2}\cdot f_{n}\cdot KIN$$

(3) $$\mathrm{STR}=-137.65-1.13\cdot 10^{-1}\cdot n\cdot 8.55\cdot 10^{-6}\cdot n^{2}+4991.51\cdot f_{n}-26944.44\cdot f_{n}{}^{2}+1.51\cdot 10^{-2}\cdot KIN+6.71\cdot 10^{-6}\;\cdot KIN^{2}-4.12\cdot 10^{-1}\cdot n\cdot f_{n}-2.16\cdot 10^{-5}\cdot n\cdot KIN+4.16\cdot 10^{-2}\cdot f_{n}\cdot KIN$$

(4) $$\mathrm{DE}=141.92+3.28\cdot 10^{-1}\cdot n\cdot 1.58\cdot 10^{-4}\cdot n^{2}-7980.27\cdot f_{n}+66814.81\cdot f_{n}{}^{2}-2.25\cdot 10^{-2}\cdot KIN+6.47\cdot 10^{-6}\;\cdot KIN^{2}-1.14\cdot n\cdot f_{n}-8.37\cdot 10^{-6}\cdot n\cdot KIN+4.59\cdot 10^{-1}\cdot f_{n}\cdot KIN$$

(5) $$R_{a}=6.93+1.87\cdot 10^{-3}\cdot n-4.99\cdot 10^{-7}\cdot n^{2}-178.55\cdot f_{n}+1332.83\cdot f_{n}{}^{2}5.65\cdot 10^{-4}\cdot KIN+9.32\cdot 10^{-8}\;\cdot KIN^{2}-2.04\cdot 10^{-2}\cdot n\cdot f_{n}-3.22\cdot 10^{-7}\cdot n\cdot KIN-3.73\cdot 10^{-3}\cdot f_{n}\cdot KIN$$

**Figure 7:**
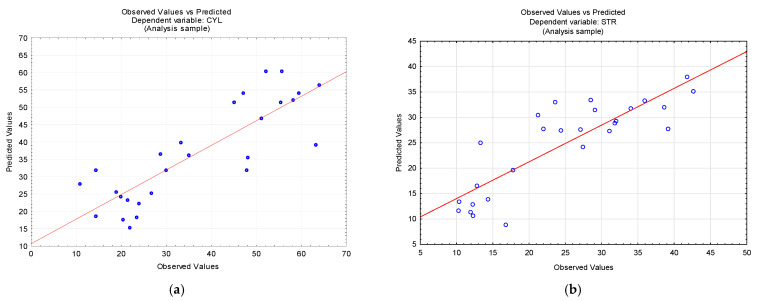

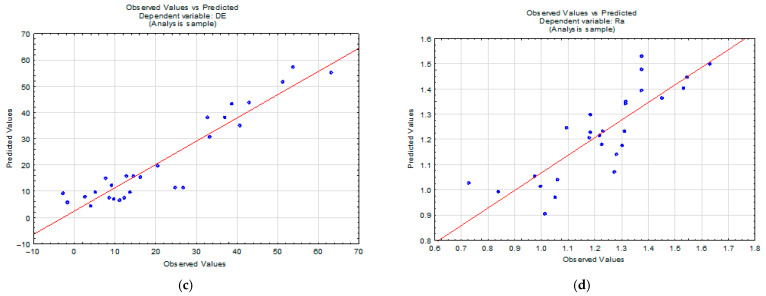
Experimental versus predicted data: (**a**) the cylindricity error; (**b**) the straightness error; (**c**) the diameter error; and (**d**) the parameter R_a_.

Figure 7 compares the experimental values with those predicted for all the output parameters. From Figure 6, it is apparent that there is a positive relationship between the data (R^2^ > 60%). The minimal differences between the predicted and experimental values are due to a large number of workpieces tested (27).

**Figure 8:**
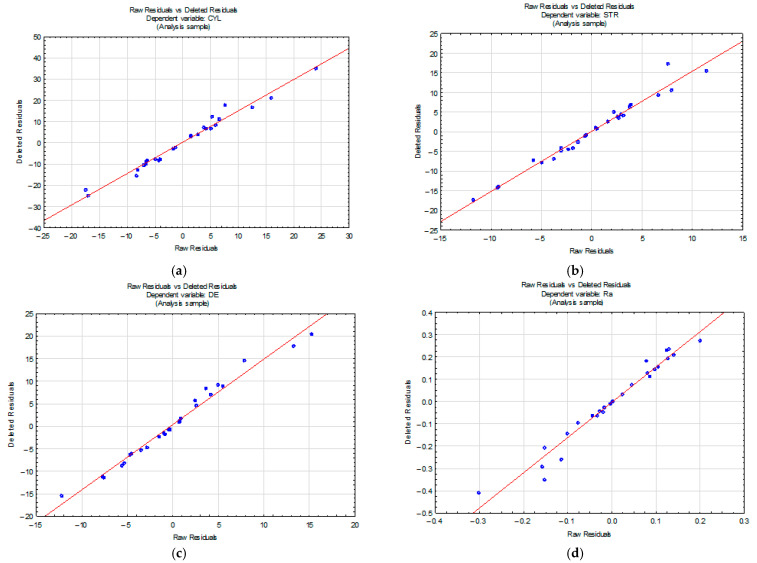
Normal probability plots for: (**a**) the cylindricity error; (**b**) the straightness error; (**c**) the diameter error; and (**d**) the parameter R_a_.

Figure 8 shows plots of residuals for all the output parameters. They confirm that the assumption of normal distribution is fulfilled because of the small scatter of points plotted along the straight line.

**Figure 9:**
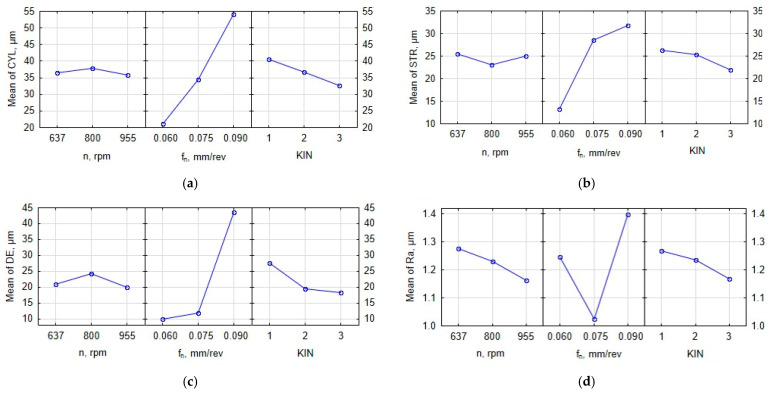
Main effects plot for: (**a**) the cylindricity error; (**b**) the straightness error; (**c**) the diameter error; and (**d**) the parameter R_a_.

Figure 9 shows the relationship between the input parameters (feed per revolution, spindle speed, and type of kinematic system) and the output parameters (CYL, STR, DE and R_a_) for through holes drilled in Inconel 718. As can be seen from Figure 9a, at a spindle speed of 955 rev/min, the cylindricity error is 35.7 µm. The most optimal value of the feed per revolution is 0.06 mm/rev; at this value, the cylindricity error (CYL_avg_) is 21.2 µm. The lowest cylindricity error of 32.5 µm was reported for the third kinematic system. For KIN I and KIN II, the parameter is higher (CYL_avg_ = 37.5 µm). Figure 9b shows that a spindle speed of 800 rev/min results in STR_avg_ = 23.0 µm. The lowest value of the straightness error (STR_avg_ = 13.1 µm) was reported at a feed per revolution of 0.06 mm/rev. The use of the third kinematic system led to the lowest value of this parameter (STR_avg_ = 21.9 µm). The first kinematic system, KIN I, was observed to be the least efficient in this respect (STR_avg_ = 26.2 µm). From Figure 9c, it is clear that the smallest dimensional accuracy of the hole was obtained at the highest spindle speed and the lowest feed per revolution. As far as the diameter error is concerned, the most efficient kinematic system was KIN III, for which DE_avg_ = 18.2 µm. The worst results were observed for the first kinematic system, where DE_avg_ = 27.5 µm. From Figure 9d, it is clear that a decrease in the spindle speed caused an increase in the parameter R_a_. Applying a feed per revolution of 0.075 mm/rev resulted in the lowest value of the parameter R_a_ (R_aavg_ = 1.023 µm). The lowest value of the parameter R_a_ was obtained for KIN III (R_aavg_= 1.167 µm). Finally, the worst results were observed for the first kinematic system, KIN I (R_aavg_ = 1.268 µm).

To sum up, the experimental data show that the third kinematic system (KIN III) was the most suitable system for drilling through holes in Inconel 718; in this case, all the four parameters describing the hole quality (CYL, STR, DE, R_a_) had the lowest values.

### 3.3. Predictive Modeling of Cylindricity Errors

**Figure 10:**
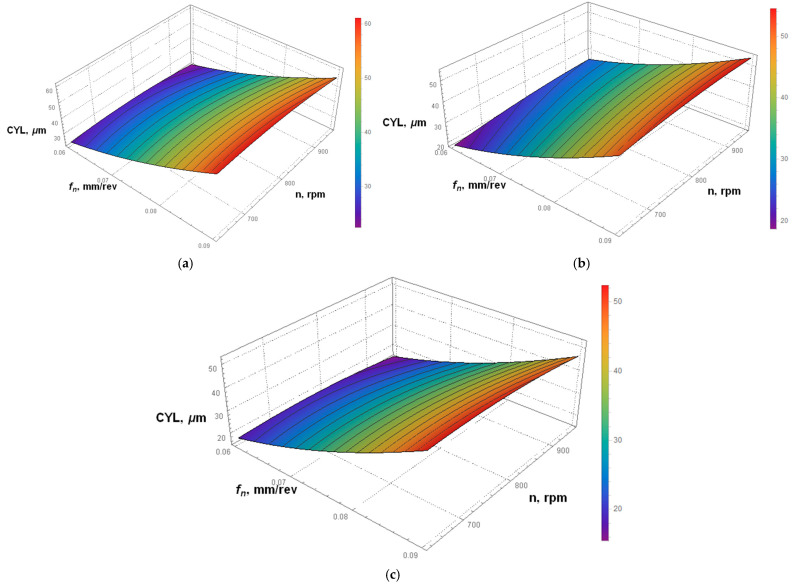
Simulations based on Equation (2); predictive modelling of the cylindricity error for: (**a**) the first kinematic system; (**b**) the second kinematic system; and (**c**) the third kinematic system.

The type of kinematic system was found to have the largest influence (61.81%) on the cylindricity error. Simulations were based on Equation (2). From Figure 10b, showing the results for the second kinematic system, it is evident that the cylindricity error is the smallest at a low feed per revolution of 0.06 mm/rev and a low spindle speed of 600 rpm. In this case, changes in the spindle speed do not have a considerable effect on the cylindricity error. However, an increase in the feed per revolution causes a rapid change in this parameter. From Figure 10a,c, illustrating the effects of the first and third kinematic systems, respectively, it is clear that the relationships are the same. A feed per revolution of 0.09 mm/rev is responsible for a higher cylindricity error. The lowest cylindricity error occurs at the lowest feed per revolution (0.06 mm/rev) and the highest spindle speed (955 rpm).

## 4. Conclusions

This article has analyzed the influence of the input parameters and kinematics of hole drilling in Inconel 718 on the output parameters describing the hole quality: diameter error; cylindricity error; straightness error; and surface texture.

The following are the conclusions drawn from the study:

- The best hole quality was obtained in the 6th (*n* = 637 rpm, *f_n_* = 0.075 mm/rev, KIN III) and 8th tests (*n* = 955 rpm, *f_n_* = 0.075 mm/rev, KIN II)
- The analysis of variance (ANOVA) proved useful to determine how the input parameters affected the hole quality
- The feed per revolution was reported to be of importance in the hole quality assessment; three out of four output parameters (STR 49.69%, DE 75.75%, R_a_ 75.36%) were dependent on it
- The type of kinematic system was the greatest contributor to the cylindricity error (CYL 61.81%)
- The regression models were characterized by high correlation values; the coefficient of determination was 70.49% for the cylindricity error, 62.13% for the straightness error, 88.50% for the diameter error and 69.73% for the parameter R_a_
- The third kinematic system was found to be the best option, as all the four output parameters (CYL, STR, DE, R_a_) reached the lowest value
- The highest efficiency of the process and the lowest values of the parameters describing the hole quality were obtained at a feed per revolution of 0.075 mm/rev

Further research will aim at determining the effects of the drilling parameters and kinematics on the roundness error, and the height and width of burrs at the hole entry and exit; it will be essential to assess the influence of each input parameter on the hole quality.

## Figures and Tables

**Table 1:** Composition of Inconel 718.

Theoretical Composition [19]						
Cu	Al.	Mo	Ni	Cr	Nb + Ta	Ti
≤0.3	0.2–0.8	2.8–3.3	50–55	17–21	4.75–5.5	0.65–1.15
Actual Composition						
Cu	Al.	Mo	Ni	Cr	Nb + Ta	Ti
0.28	0.52	2.89	52.59	20.51	4.81	0.96

**Table 2:** Mechanical properties of Inconel 718 [19].

Hardness, HB	Ultimate Tensile Strength, Rm	Yield Strength, Re	Young’s Modulus, E
363	930 MPa	550 MPa	204.9 GPa

**Table 3:** Three kinematic systems.

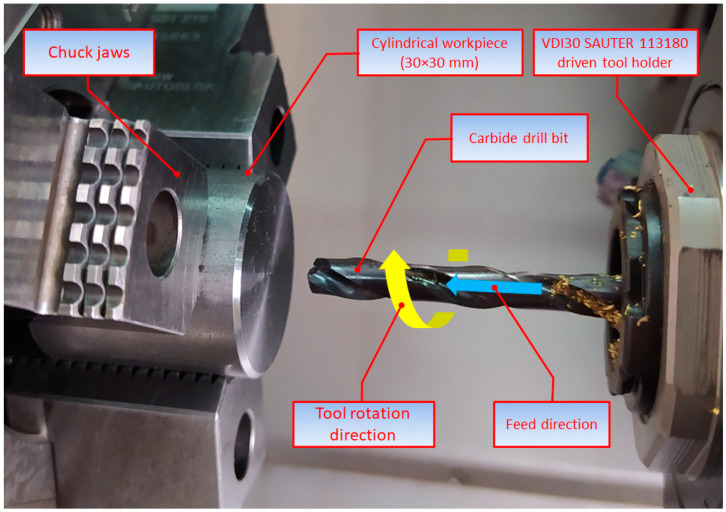	KIN I: the workpiece being stationary and the tool performing both the primary (rotary) and secondary (linear) motions
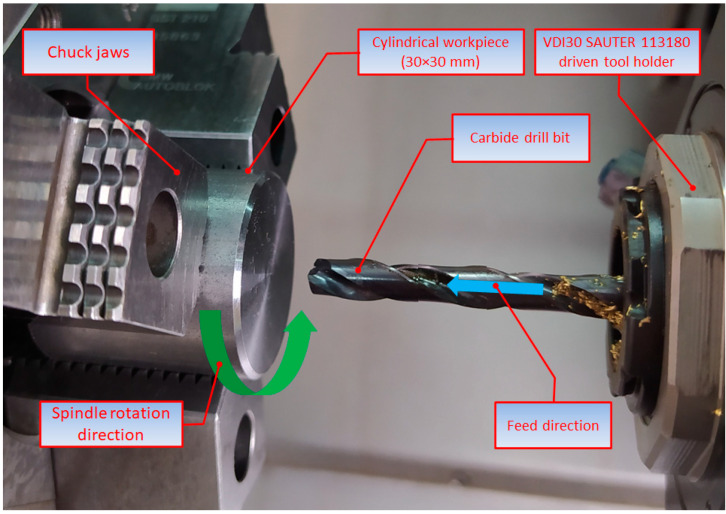	KIN II: the workpiece performing the primary (rotary) motion and the tool moving linearly, parallel to the axis of rotation of the workpiece
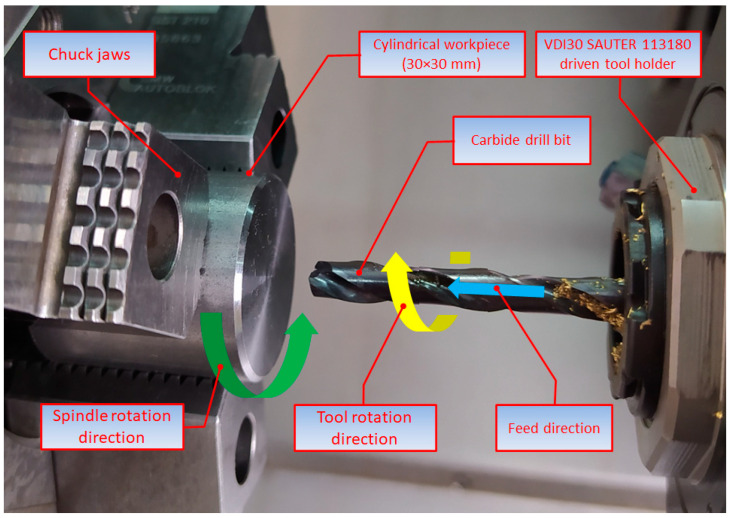	KIN III: the workpiece and the tool rotating in opposite directions with the tool performing also a linear forward motion.

**Table 4:** Parameters of the drill bit used.

Specification	
Cutting edge diameter	6 mm
Coating	TiAlNPlus
Tool holding device Type	HA parallel shank HPC UNI
Coolant supply	Internal
Chip flute length Point angle	144 m 140°
DIN	6537

**Table 5:** Input parameters used in the drilling experiments.

Experiment No.	*n*, rev/min	*f_n_*, mm/rev	Kinematic System, Equation (1)
1	800	0.075	800
2	800	0.075	−800
3	800	0.075	0
4	637	0.075	637
5	637	0.075	−637
6	637	0.075	0
7	955	0.075	955
8	955	0.075	−955
9	955	0.075	0
10	800	0.06	800
11	800	0.06	−800
12	800	0.06	0
13	637	0.06	637
14	637	0.06	−637
15	637	0.06	0
16	955	0.06	955
17	955	0.06	−955
18	955	0.06	0
19	800	0.09	800
20	800	0.09	−800
21	800	0.09	0
22	637	0.09	637
23	637	0.09	−637
24	637	0.09	0
25	955	0.09	955
26	955	0.09	−955
27	955	0.09	0

**Table 6:** ANOVA results for the cylindricity error.

Source	SS	DF	MS	F Value	*p* Value	PC
Model	5341.9610	9	593.5512	4.5113	0.0037	—
Constant	2.8470	1	2.8468	0.0216	0.8848	—
*n*	26.2140	1	26.2145	0.1992	0.6610	5.77
*n* ^2^	24.7210	1	24.7214	0.1879	0.6701	5.44
*f_n_*	9.4190	1	9.419	0.0716	0.7923	2.07
*f_n_* ^2^	68.0070	1	68.0067	0.5169	0.4819	14.98
KIN	14.9900	1	14.99	0.1139	0.7398	3.30
KIN^2^	230.6290	1	230.6289	1.7529	0.2030	50.79
*n* · *f_n_*	7.1700	1	7.1696	0.0545	0.8182	1.58
*n*·KIN	56.3450	1	56.3448	0.4283	0.5216	12.41
*f_n_*·KIN	13.7840	1	13.7839	0.1048	0.7501	3.04
Error	2236.6660	17	131.5686	—	—	29.51
Total	7578.6270	26	—	—	—	100.00

Multiple R = 0.8395; Multiple R^2^ = 0.7049; Adjusted R^2^ = 0.5486.

**Table 7:** ANOVA results for the straightness error.

Source	SS	DF	MS	F Value	*p* Value	PC
Model	1850.6160	9	205.6240	3.0992	0.0214	—
Constant	49.1180	1	49.1175	0.7403	0.4015	—
*n*	2.5320	1	2.5318	0.0382	0.8474	0.52
*n* ^2^	6.6170	1	6.6170	0.0997	0.7560	1.36
*f_n_*	147.0860	1	147.0861	2.2169	0.1548	30.12
*f_n_* ^2^	88.1670	1	88.1667	1.3289	0.2650	18.06
KIN	15.9760	1	15.9759	0.2408	0.6299	3.27
KIN^2^	121.6150	1	121.6151	1.8330	0.1935	24.91
*n* · *f_n_*	11.7140	1	11.7141	0.1766	0.6796	2.40
*n*·KIN	42.4170	1	42.4165	0.6393	0.4350	8.69
*f_n_*·KIN	3.0550	1	3.0552	0.0460	0.8326	0.63
Error	1127.9040	17	66.3473	—	—	37.87
Total	2978.5200	26	—	—	—	100.00

Multiple R = 0.7882; Multiple R^2^ = 0.6213; Adjusted R^2^ = 0.4208.

**Table 8:** ANOVA results for the diameter error.

Source	SS	DF	MS	F Value	*p* Value	PC
Model	7481.2380	9	831.2487	14.5361	0.0000	—
Constant	79.5490	1	79.5490	1.3911	0.2545	—
*n*	144.5960	1	144.5960	2.5286	0.1302	4.67
*n* ^2^	96.1960	1	96.1960	1.6822	0.2120	3.10
*f_n_*	761.2260	1	761.2260	13.3116	0.0020	24.56
*f_n_* ^2^	1356.0070	1	1356.0070	23.7126	0.0001	43.75
KIN	70.3140	1	70.3140	1.2296	0.2829	2.27
KIN^2^	118.0150	1	118.0150	2.0637	0.1690	3.81
*n* · *f_n_*	89.9380	1	89.9380	1.5728	0.2268	2.90
*n*·KIN	12.4230	1	12.4230	0.2173	0.6471	0.40
*f_n_*·KIN	371.0610	1	371.0610	6.4888	0.0208	11.97
Error	972.1480	17	57.185	—	—	11.50
Total	8453.3860	26	—	—	—	100.00

Multiple R = 0.9407; Multiple R^2^ = 0.8850; Adjusted R^2^ = 0.8241.

**Table 9:** ANOVA results for the parameter R_a_.

Source	SS	DF	MS	F Value	*p* Value	PC
Model	0.8014	9	0.0890	4.3502	0.0044	—
Constant	0.1901	1	0.1901	9.2880	0.0072	—
*n*	0.0046	1	0.0046	0.2294	0.6380	0.37
*n* ^2^	0.0009	1	0.0009	0.0465	0.8316	0.08
*f_n_*	0.3812	1	0.3812	18.6235	0.0004	30.33
*f_n_* ^2^	0.5396	1	0.5396	26.3602	0.0000	42.92
KIN	0.0443	1	0.0443	2.1678	0.1591	3.53
KIN^2^	0.0244	1	0.0244	1.1956	0.2894	1.95
*n* · *f_n_*	0.0285	1	0.0285	1.3927	0.2542	2.27
*n*·KIN	0.0185	1	0.0185	0.9085	0.3538	1.48
*f_n_*·KIN	0.0245	1	0.0245	1.1981	0.2889	1.95
Error	0.3479	17	0.0204	—	—	30.27
Total	1.1495	26	—	—	—	100.00

Multiple R = 0.8350; Multiple R^2^ = 0.6972; Adjusted R^2^ = 0.5370.

## Data Availability

Data Sharing is not applicable.

## Nomenclature

n	spindle speed, rpm
*n_n_*	tool speed, rpm
$f_{n}$	feed per revolution, mm/rev
*KIN*	kinematics
SS	sum of squares
DF	degrees of freedom
MS	mean square
CYL	cylindricity error, µm
STR	straightness error, µm
DE	diameter error, µm
UPR	undulations per revolution
λc	wavelength
*p*	significance
PC	percentage contribution
LCO_2_	liquid carbon dioxide
LN_2_	liquid nitrogen
